# Effects of Green cardamom (*Elettaria cardamomum* Maton) and its combination with cyclophosphamide on Ehrlich solid tumors

**DOI:** 10.1186/s12906-021-03305-2

**Published:** 2021-04-29

**Authors:** Rafa S. Almeer, Meshael Alnasser, Nada Aljarba, Gadah I. AlBasher

**Affiliations:** 1 Department of Zoology, College of Science, King Saud University, Riyadh, Saudi Arabia; 2 Biology Department, Faculty of Science, Princess Nourah bint Abdulrahman University, Riyadh, Saudi Arabia

**Keywords:** Cardamom, Cyclophosphamide, Anticancer, Toxicity, EST-bearing mice

## Abstract

**Background:**

Cardamom (*Elettaria cardamomum*) is a spice and exhibits potent antioxidant and biological activities through distinct molecular mechanisms. However, the anticancer effect of cardamom was not explored yet in Ehrlich solid tumor (EST)-bearing mice.

**Objectives:**

This investigation was aimed to evaluate the anti-cancer effects of green cardamom (GCar) alone or combined with the anti-cancer drug cyclophosphamide in an in vivo model to explore its mechanistic role in tumor cell death in EST-bearing mice.

**Methods:**

Ehrlich ascites tumor cells were injected in the mice and 5 days later the animals treated with GCar and/or cyclophosphamide for 10 days. Twenty-four hours from the last treatment, animals were sacrificed for the different measurements.

**Results:**

Data recorded for tumor size, percentage of tumor growth inhibition, tumor growth delay and mean survival time of EST-bearing mice demonstrated the effective role of GCar alone or combined with CPO as a promising anti-cancer agent because it reduced tumor size. GCar elevated the mean survival time of EST-bearing mice compared to that of untreated EST and EST + CPO groups. Analysis of qPCR mRNA gene and protein expression revealed that GCar alone or combined with CPO were promising anticancer agents. After the treatment of EST with GCar, the apoptotic-related genes and proteins were significantly modulated. GCar induced markedly significant decreases in oxidative stress biomarkers and a significant increment in glutathione levels and that of antioxidant enzymes. With a marked diminish in liver and kidney function biomarkers.

**Conclusion:**

The results revealed that GCar could serve as an apoptotic stimulator agent, presenting a novel and potentially curative approach for cancer treatment, inducing fewer side effects than those of the commercially used anti-cancer drugs, such as CPO.

**Supplementary Information:**

The online version contains supplementary material available at 10.1186/s12906-021-03305-2.

## Background

Chemotherapy is one of the cancer treatment approaches, which may be undertaken alone or together with other therapies, such as surgery and/or radiotherapies. It utilizes a wide range of remedies that have cytotoxic activities to one or more cancerous tissues. Cyclophosphamide (CPO) is an alkylating cytotoxic agent that has been effectively used in patients for the last century for the curing of neoplastic maladies, such as solid tumors and lymphomas, as well as non-neoplastic maladies, such as rheumatoid arthritis and systemic lupus erythrematosus [1]. However, the medical use of CPO has been inadequate because of its capability to harm normal tissues, which usually results in toxicity in several organs (heart, testes and urinary bladder) [2] Hepatotoxicity is the foremost side effect of CPO because it is metabolized primarily within the hepatocytes by hepatic microsomal cytochrome p450 varied function oxidase system to construct its two-active metabolites phosphoramide mustard (PM) and acrolein. Phosphoramide is linked to the immunosuppressive and antineoplastic outcomes of CPO, while acrolein is associated with the toxic effects of CPO. Studies have indicated that oxidative damage is linked with its hepatotoxic effects [3]. CPO toxicity resulting from acrolein binding to the nucleophilic cellular antioxidants, such as glutathione (GSH), results in the exhaustion of the antioxidant defiance molecules and also initiates lipid peroxidation (LPO) [2, 4].

In recent years, many herbal byproducts and phytomedicines have drawn the attention of researchers mainly because of convincing anticancer activities with insignificant side effects [5]. Cardamom (*Elettaria cardamomum*) is a spice found in the form of tiny pods with dark seeds. The seeds have strongly sweet and aromatic tastes. Both the seed and the pod have a wealthy smell and are often used in desserts, hot and spicy plates, as well as aromatic beverages, coffees and teas. It is considered as the king of spices and is one of the most luxurious spices, ranking third, with the first and second being saffron (derived from the flower of *Crocus sativus*) and vanilla (derived from orchids of the genus Vanilla), respectively [6]. Cardamom is a multi-use spice. It was utilized by ancient Egyptians for its medicinal properties and even for breast-cancer anticipation [7]. The pleasant odor profile is an fundamental constituent of cardamom essential oil, the major ingredients of cardamom essential oil are pinene, myrcene, methyl eugenol, 1,8-cineole, a-terpinyl acetate, sabinene, phellandrene, terpinene, limonene, p-cymene, terpinolene, linalool, linalyl acetate, terpinen-4-ol, nerol, citronellol, geraniol, and trans-nerolidal [8]. Cardamom has also been used for teeth, gum and throat infection, as well as against lung congestion, pulmonary tuberculosis and digestive disorders [9]. Cardamom was found to reduce weight gain and associated metabolic damage to the body [10]. Cardamom suppressed edema to a lesser extent, whereas a higher dose of cardamom reveals a more potent anti-inflammatory effect on the skin in the presence of the reference drug, indomethacin [11]. The chemopreventive effect of cardamom has also been demonstrated earlier to control colorectal cancer [12] and to prevent papillomagenesis on the skin [9]. Furthermore, cardamom extract was found to lessen PTSD-like anxiety symptoms in test animals [13]. One study found that when cardamom was consumed during pregnancy, offspring showed enhanced learning, memory, and behavior [14].

The Ehrlich tumor is a transplantable neoplasm, which corresponds to mammary adenocarcinoma in female. When implanted intraperitoneally, it grows in the ascitic form and when implanted subcutaneously, it grows into a solid tumor. This tumor has been used to grow a tumor model [15]. Ehrlich ascites carcinoma (EAC) or Ehrlich solid tumor (EST) model has basically been used for study of tumor pathogenesis and development of anti-tumorigenic agents. In this context, El-Garawani et al. [5] and Salah et al. [16] were used EAC to study the anticancer effect of *Candelariella vitellina*, and B12 and sitagliptin, respectively.

The aim of this investigation is to examine the possible anticancer efficacies of green cardamom (GCar) alone or in combination with CPO on Ehrlich solid tumor-bearing mice. In addition, the study will extend to examine the protective effects of GCar against CPO-induced hepatotoxicity and nephrotoxicity and determine whether this effect will be modulated through an antioxidant mechanism in the liver and kidney.

## Methods

### Plant material

The seeds of GCar were collected from a local market in Riyadh, Saudi Arabia, in 2019, and were identified and authenticated by a taxonomist from Department of Botany, College of Science, King Saud University. The seeds were powdered with an electrical grinder and then extracted three times by maceration with 70% methanol. The solvent was evaporated using a vacuum evaporator and then lyophilized to remove water [17]. The GCar extract was stored at − 20 °C until used. The obtained extract was evaluated with HPLC to determine the phenolics and flavonoids constituents of GCar. The HPLC condition was previously reported by Dkhil et al. [18]. The HPLC fingerprint of the GCar revealed the presence of many phenolics and flavonoids compounds, possibly ferulic acid and its derivatives, luteolin, rutin, catechin, caffeic acid, gallic acid and its derivatives, quercetin, and cinnamic acid (Supplementary data; Table S1).

### Ehrlich ascites tumor (EAT) cells

The EAT cell line was sourced from the American Type Tissue Culture Collection (Manassas, VA, USA). EAT cells were counted using a Neubauer hemocytometer with the trypan blue dye exclusion procedure. EAT cells (25 × 10^5^/mouse) were diluted with 0.2 mL of physiological sterile saline solution (0.9% NaCl) and injected intramuscularly (i.m.) in the right thigh of male BALB/c mice.

### Doses of cardamom and CPO

The dose of cardamom given in this study was 100 mg kg^− 1^ body weight according to a previous study on the toxicity of this plant by Goyal et al. [19]. However, the selected dose of CPO was 50 mg kg^− 1^ body weight on the basis of a previous study by Twelves et al. [20].

### Experimental animals

Male Swiss albino mice (6–8 weeks old and weighing 23 ± 4 g) were obtained from Biology Department of Princess Nourah bint Abdulrahman University (PNU; Riyadh, Saudi Arabia). Mice were housed under standard laboratory conditions and provided with water and a normal standard basic diet. Mice were acclimatized to experimental conditions for seven-days prior to the experiments. All experimental protocols were in accordance with the handling and care of animals at Princess Nourah bint Abdulrahman University (Approval number: H-01-R-059/19–0272, Riyadh, Saudi Arabia). All procedures of this study adhere to the ARRIVE Guidelines for reporting animal research. On day zero, all the mice were injected i.m. with EAT cells and then, on the day four, mice were divided at random into six experimental groups (10 mice per group), as follows:

1. EST untreated control group (Con): Mice were injected intraperitoneally (i.p.) with 0.2 ml of physiological saline solution on the 10th day post tumor inoculation (PTI).
2. EST + CPO treated group (CPO): Mice were injected i.p. with a single dose of CPO equal to 50 mg kg^− 1^ body weight on the 10th day PTI for 3 consecutive days.
3. EST + green cardamom treated group (GCar): Mice were administered orally once daily with GCar at a dose equal to 100 mg kg^− 1^ body weight for 10 days starting on day 5 PTI.
4. EST + green cardamom+CPO combined treated group (GCar-CPO): Mice received an oral dose equal to 100 mg kg^− 1^ body weight of GCar daily for 5 days starting on day 5, on the 10th day PTI they were injected i.p. with a single dose of CPO equal to 50 mg kg^− 1^ body weight and then they receive a single oral dose equal to 100 mg kg^− 1^ body weight of GCar daily for another 5 days.

The experimental design was followed our previous study [15]. Twenty-four hours from the last treatment (day 16 PTI), seven animals of each group were sacrificed by intraperitoneal injection of pentobarbital (200 mg kg^− 1^) and three animals from each group were left to calculate survival time, tumor growth delay time and inhibition of tumor growth.

### Body weight (BW) changes

Changes in BW were estimated as follows: initial BW was that on day 12 PTI. Final and net final BWs were estimated on day 20 PTI. Net final BW = final BW – tumor weight. Percentage difference of BW gain was determined as BW gain ([final BW – initial BW]/initial BW) × 100. Tumor mass (mg) was determined via the following equation, as demonstrated by Jaganathan et al. [21]:

$$ \mathrm{Tumor}\ \mathrm{mass}=\frac{\mathrm{Length}\ \left(\mathrm{mm}\right)\times {\left[\mathrm{Width}\ \left(\mathrm{mm}\right)\right]}^2}{2} $$

### Tumor volume

Tumor volume was determined using the following equation, as mentioned previously [15]:

$$ \mathrm{Tumor}\kern0.17em \mathrm{volume}\left(\mathrm{V}\right)=4/3\prod {\mathrm{r}}_1^2\times {\mathrm{r}}_2{\left(\mathrm{cm}\right)}^3 $$

Where, *r*_1_ and *r*_2_ are the radii of tumors in two different planes determined with Vernier calipers.

### Evaluation of antitumor activity

The tumor growth inhibition ratio (T/C %) was estimated on day 21 by comparing the average values for the treated and untreated tumor-bearing mice. Tumor growth in untreated EST mice was considered to be 100%.

$$ \mathrm{T}/\mathrm{C}\%=\frac{\left(\mathrm{average}\ \mathrm{tumor}\ \mathrm{mass}\ \mathrm{of}\ \mathrm{EST}\ \mathrm{mice}-\mathrm{average}\ \mathrm{tumor}\ \mathrm{mass}\ \mathrm{of}\ \mathrm{treated}\ \mathrm{mice}\right)\times 100}{\mathrm{Average}\ \mathrm{tumor}\ \mathrm{mass}\ \mathrm{of}\ \mathrm{EST}\ \mathrm{mice}} $$

### Percentage increase in life span (ILS)

Mean survival time (MST; *n* = 3/group) was estimated by monitoring mortality on a daily basis and the % ILS was estimated via the following equation, as reported previously [15]:

$$ \mathrm{MST}=\left(\mathrm{day}\ \mathrm{of}\ \mathrm{first}\ \mathrm{death}+\mathrm{day}\ \mathrm{of}\ \mathrm{last}\ \mathrm{death}\right)/2 $$

$$ \%\mathrm{ILS}=\left(\mathrm{MST}\ \mathrm{of}\ \mathrm{treated}\ \mathrm{group}/\mathrm{MST}\ \mathrm{of}\ \mathrm{EST}-\mathrm{untreated}\ \mathrm{group}\right)\times 100 $$

### Gene expression analysis

Apoptosis controlled genes expression such as Bcl2, Bax and caspase-3, − 8 and − 9, was estimated utilizing β-actin as a reference housekeeper gene. RNA was extracted from the EST samples with TRIzol Reagent (Invitrogen, Carlsbad, CA, USA). Reverse Transcription System (Promega, Madison, WI, USA) was used to prepare the cDNA. Real-time PCR was performed using SYBR Green Master Mix (Applied Biosystems) on the Applied Biosystems StepOne™ real-time PCR system (Applied Biosystems, Carlsbad, CA, USA). The PCR was performed as follows: incubation at 95 °C for 10 min followed by 40 cycles for 15 s at 94 °C, 30 s at 58 °C and 30 s at 70 °C. The Pfaffl formula was used for data analysis utilizing the following equation: 2^−ΔΔCt^ [22]. The primer sets were as follows:

β-actin F: 5′-CCA-CCA-TGT-ACC-CAG-GCA-TT-3′.

R: 5′-AGG-GTG-TAA-AAC-GCA-GCT-CA-3′.

Bcl-2 F: 5′-ACA-GGG-TAC-GAT-AAC-CGG-GA-3′.

R: 5′-GCC-CAG-ACT-CAC-ATC-ACC-AA-3′.

Bax F: 5′-GAC-ATT-GGA-CTT-CCT-CCG-GG-3′.

R: 5′-ACA-GGG-ACA-TCA-GTC-GCT-TC-3′.

Caspase-3 F:5′-GCG-GTT-GTA-GAA-GTT-AAT-AAA-GGT-A-3′.

R: 5′-AAC-CAG-GTG-CTG-TGG-AGT-AT-3′.

Caspase-8 F: 5′-GGT-TAG-GGG-ACT-CGG-AGA-CT-3′.

R: 5′-CAG-GCT-CAG-GAA-CTT-GAG-GG-3′.

Caspase-9 F: 5′-TTG-GTG-ATG-TCG-AGC-AGA-AAG-A-3′.

R: 5′-GGG-ACA-CAA-GTC-ACT-AGC-CC-3′.

### Liver functions parameters

The serum levels of alanine transferase (ALT) and aspartate transferase (AST) were analyzed with a spectrophotometric method [23]. Total bilirubin (TB) in serum was assayed using the previously described method [24].

### Kidney function parameters

The levels of kidney function markers (urea and creatinine) in serum were measured using commercial kits purchased from Cayman Chemical (Ann Arbor, Michigan, USA) and the procedures were performed according to the manufacturer’s protocols.

### Oxidative stress biomarkers and antioxidant enzyme activities

Malondialdehyde (MDA) amounts, an indicator for LPO, were assayed in EST tissue homogenates by reaction with thiobarbituric acid [25]. Similarly, the homogenates were used to quantify nitric oxide (NO) level [26] using Griess reagent and reduced glutathione (GSH) content [27] by using Ellman’s (5,5′-dithiobis-(2-nitrobenzoic acid) or DTNB) reagent. Whereas, glutathione peroxidase (GPx), glutathione reductase (GR), superoxide dismutase (SOD), and catalase (CAT) were assayed using specific commercial kits obtained from Cayman Chemical and assays procedures were followed the supplier’s protocols.

### Histopathology and immunohistochemistry analyses

Pieces of tumor samples were fixed in 4% neutral paraformaldehyde for 24 h. Hematoxylin and eosin (H&E) staining for light microscopy was applied. For the recognition of apoptosis controlled proteins, the prepared sections (5 μm thickness) were blocked with 0.1% H_2_O_2_ for 15 min. Then, the specimens were incubated with rabbit polyclonal Bcl-2, Bax or caspases-3 antibody at 4 °C during the night. The specimens were rinsed with PBS, incubated with biotinylated goat anti-rabbit immunoglobulins and then with streptavidin–peroxidase complexes at 30 °C for half an hour. The peroxidase activity was developed with diaminobenzidine (DAB)-hydrogen peroxide. Specimens were captured at 400× magnification.

### Statistical analysis

Data were represented as the means ± standard deviations (SD). Statistical significance was determined by one-way analysis of variance (ANOVA), followed by Duncan’s post hoc test. A *p* value < 0.05 was considered statistically significant.

## Results

### BW and survival of mice

**Fig. 1:**
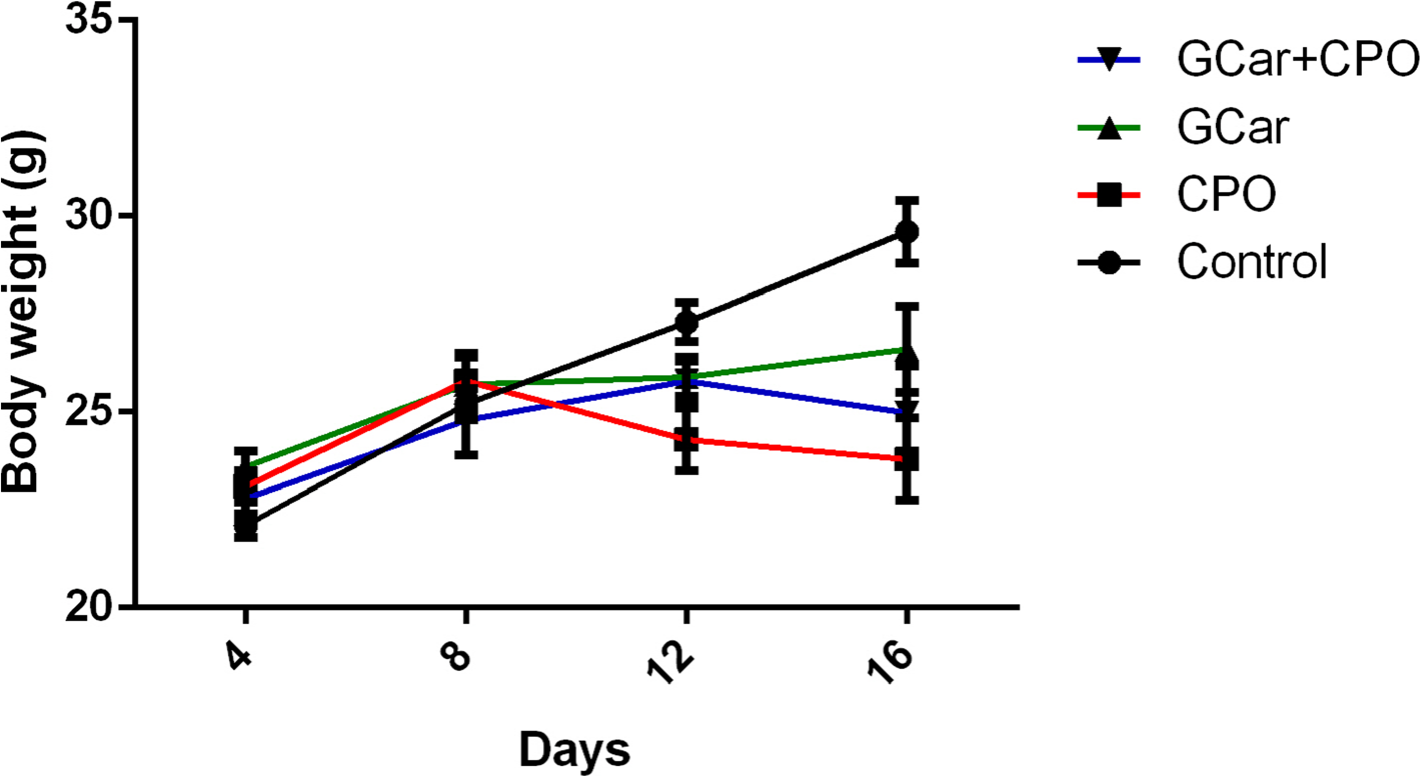
Effects of GCar, CPO and GCar-CPO on body weights of the different treatment groups

As shown in Fig. 1, EST-inoculated mice treated with GCar, CPO, or GCar-CPO showed a marked (*p* < 0.05) decline in BW when compared with that of untreated EST mice. The greatest decline in BW was showed in the CPO-treated mice.

**Fig. 2:**
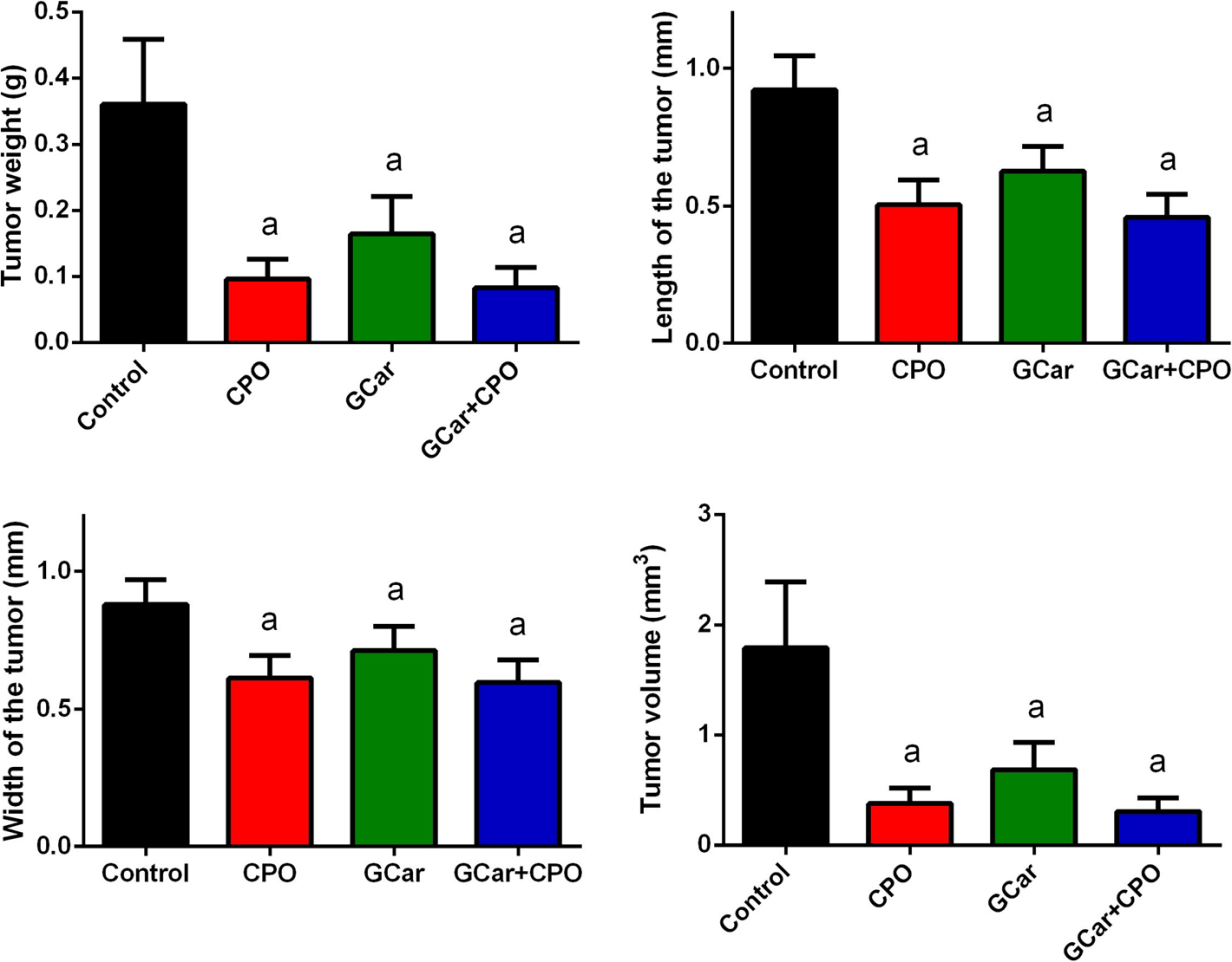
Effects of CPO, GCar and GCar-CPO on the tumor weight, length, width of the tumor and tumor volume. Values are expressed as means ± SD. a and b: Significant against control and CPO groups at *p* < 0.05, respectively

The results indicated that administration of tumor-bearing EST mice with GCar or CPO alone, or in combination markedly decreased tumor development, as recognized by the reduction in tumor volume and tumor weight, compared with those in the tumor-bearing EST mice (Fig. 2).

**Fig. 3:**
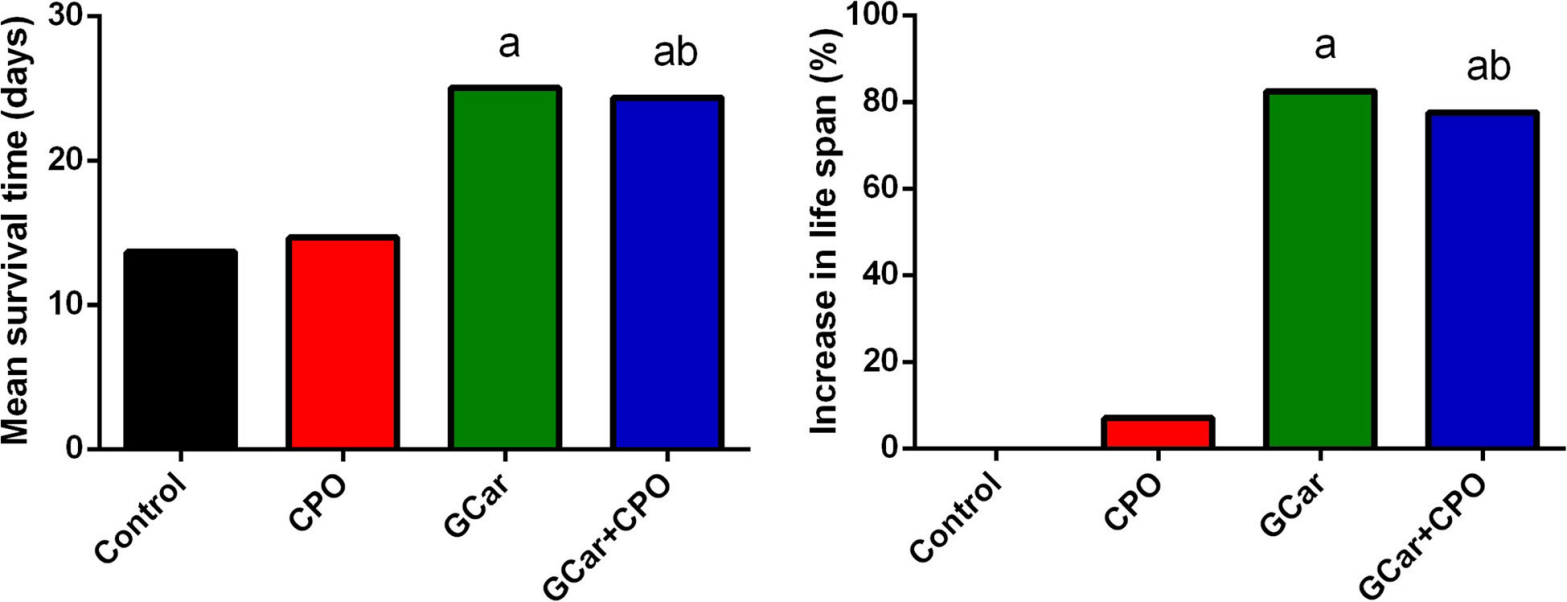
Effects of CPO, GCar and GCar-CPO on the mean survival time (days) and increase of life span ratio (ILS%). Values are expressed as means ± SD. a and b: Significant against control and CPO groups at *p* < 0.05, respectively

Interestingly, we found that MST for the control EST-bearing mice and CPO-treated mice were 13.7 and 14.6 days, respectively, which increased to 25 and 24.3 days in the GCar and GCar-CPO groups, respectively (Fig. 3). Furthermore, ILS for the GCar and GCar-CPO groups increased significantly in comparsion with that for the CPO-treated mice and untreated EST-bearing mice.

### Histopathology of EST in mice

**Fig. 4:**
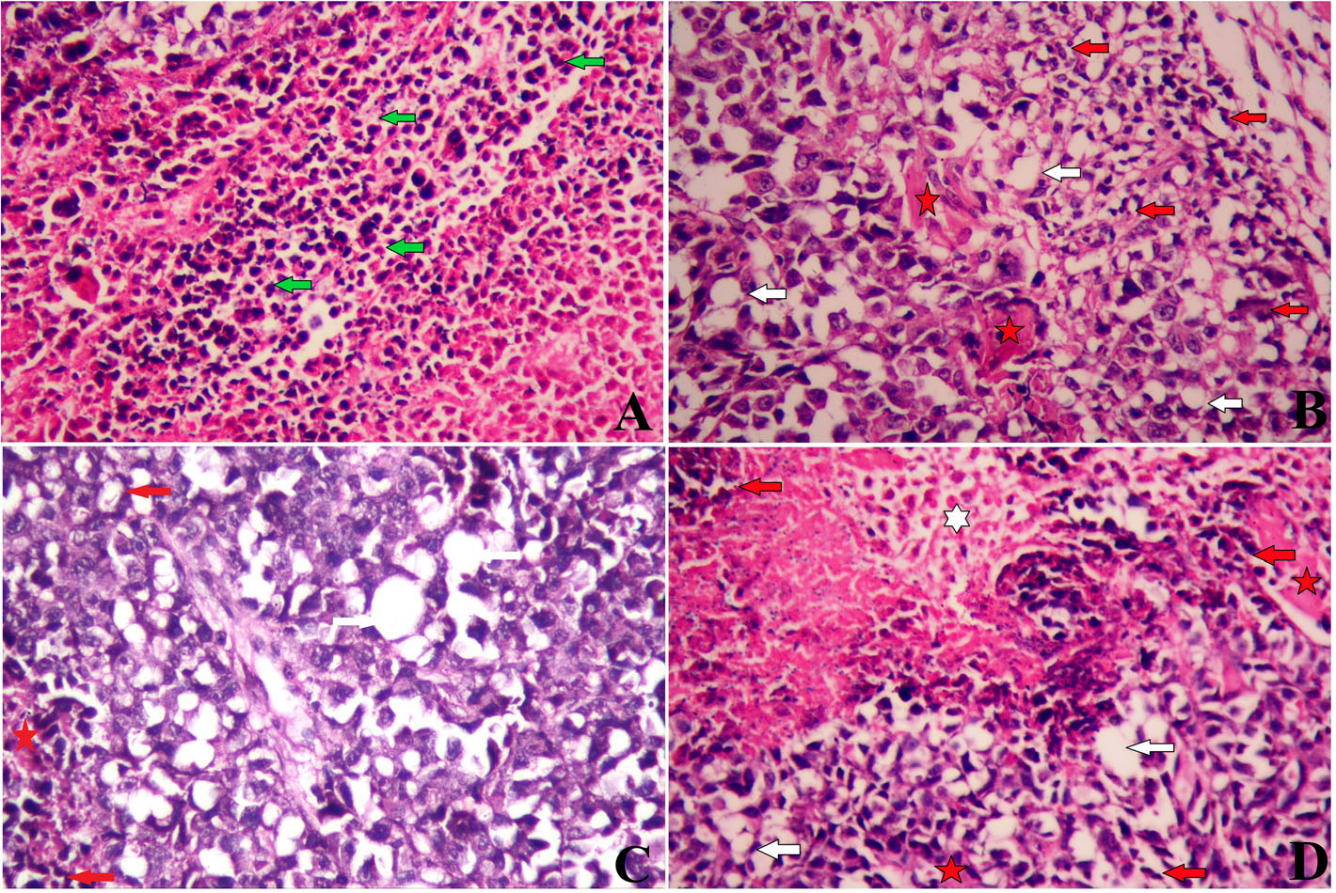
Muscle fibers between destructed tumor cells (red arrow), fat vacuoles in between tumor cells (white arrow) and remnants of dead tumor cells (apoptotic cells: red arrow; necrotic cells: white star) in the treated mice; compaction and aggregation of the large, round and polygonal tumor cells (green arrow). **a** Control EST, Control EST section shows compaction and aggregation of the large, round and polygonal tumor cells. **b** CPO, **c** EST + GCar and **d** EST + GCar-CPO. All magnifications at × 400

Healthy mice inoculated with EST cells grew tumor at the site of injection. Figure 4a, indicated that slides from EST-bearing mice demonstrated numerous cellular and nuclear pleomorphisms, as could be seen by the compacted and combined tumor cells extend within the muscles tissue, as well as groups of large, round, and polygonal cells with pleomorphic shapes, binucleation, and hyperchromatic nuclei. However, EST-bearing group cured with CPO showed some necrotic areas and the remnant cancer cells encircled the muscles tissue (Fig. 4b). More demolition of EST tissue was found after the oral administration of GCar alone (Fig. 4c) or in combination with CPO (Fig. 4d). In these groups, the tumor was sporadic and appeared to be developed slowly and fragmented. Our findings demonstrated that GCar had partial anti-cancer activity.

### qPCR data and immunohistochemistry results

**Fig. 5:**
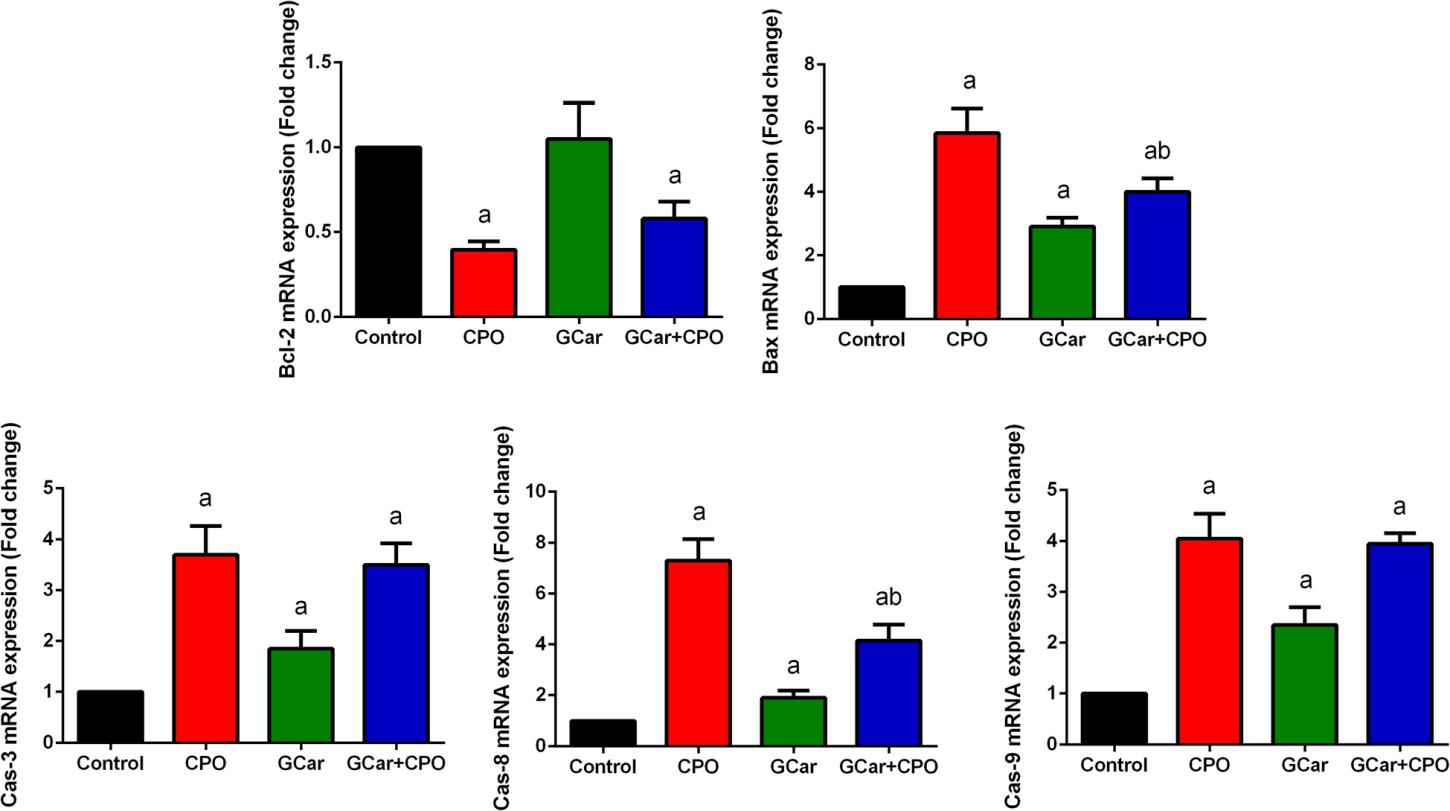
Effects of CPO, GCar and GCar-CPO on the mRNA expression of Bcl-2, Bax, caspase-3, caspase-8 and caspase-9 in the tumor tissue. Values are expressed as means ± SD of triplicate experiments. a and b: Significant against control and CPO groups at *p* < 0.05, respectively

The data represented in Fig. 5 indicated a significant downregulation in mRNA of Bcl-2 in CPO-treated EST-bearing groups in comparison with that of the control EST-bearing mice. Furthermore, EST-bearing mice treated with GCar mixed with CPO showed a considerable drop in mRNA expression of Bcl-2 at *p* < 0.05 versus that of CPO treatment alone. Conversely, a highly marked up-regulation was observed in Bax mRNA expression in mice treated with GCar, CPO and GCar-CPO in comparison with that in the control EST-bearing group (Fig. 5). Moreover, highly marked increments were exhibited in mRNA expression for caspase-3, − 8 and − 9 in mice treated with GCar, CPO and GCar-CPO versus that of the control EST-bearing group (Fig. 5).

**Fig. 6:**
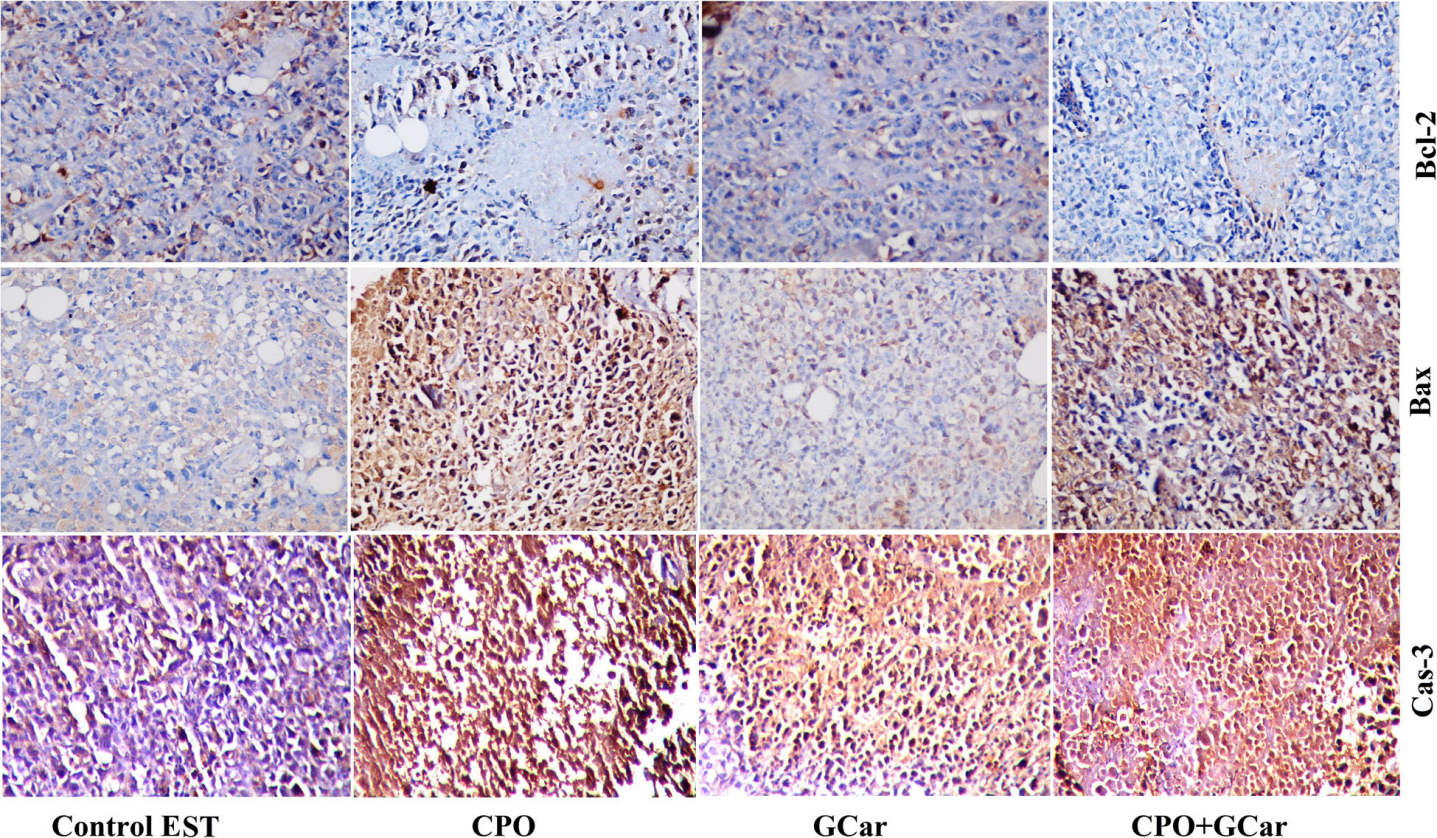
Figures show apoptotic proteins immunoreactivity in tumor tissues obtained from mice. All magnifications at × 400

Immunohistochemistry analysis was conducted to confirm the effects of GCar on apoptosis-related proteins. The results revealed that GCar administration induced up-regulation of Bax and caspase-3, and down-regulation of Bcl-2 compared to that of the EST-bearing group (Fig. 6). Furthermore, as expected, CPO treatment induced up-regulation of Bax and caspase-3, and down-regulation of Bcl-2 compared to that of the EST-bearing group. The same effects were found in the GCar-CPO treated group.

### Oxidative stress biomarkers

**Fig. 7:**
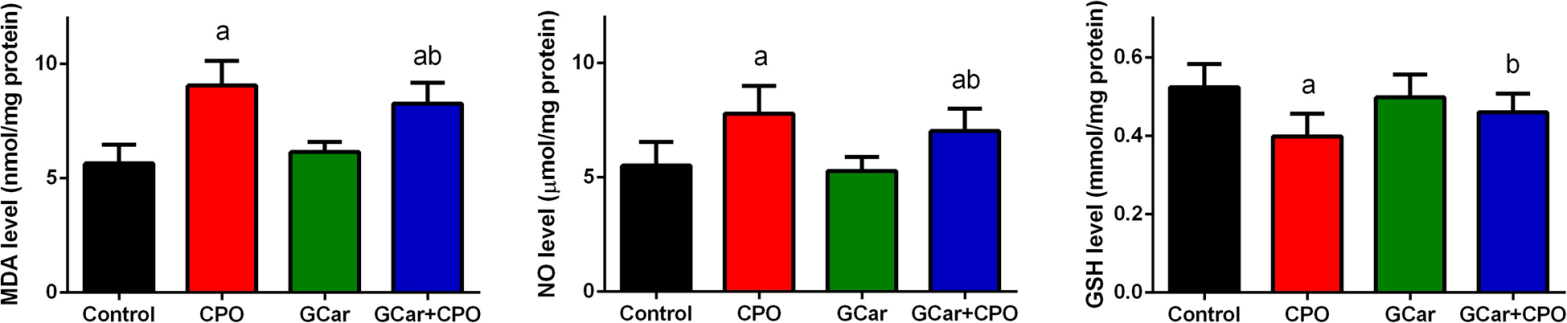
Effects of CPO, GCar and GCar-CPO on the LPO, NO and GSH levels in the tumor tissue. Values are expressed as means ± SD. a and b: Significant against control and CPO groups at *p* < 0.05, respectively

As expected, a marked elevation in MDA level was found in the CPO-treated group, compared to that in the EST-bearing mice (Fig. 7). However, MDA levels markedly declined in the combination treatment group, compared with that in the CPO group, demonstrating the antioxidant effect of cardamom. In addition, a marked elevation in NO levels was shown in the CPO-treated mice compared with that in the EST-bearing mice. Conversely, NO levels declined non-significantly in the GCar group, but significantly increased in the GCar-CPO group compared to that in the EST-bearing mice. GSH levels decreased in the GCar group compared with that in the EST-bearing mice; however, this decrease did not achieve statistical significance. A marked diminishes in GSH levels was observed in the CPO and GCar-CPO-treated mice compared with that in the EST-bearing mice (Fig. 7).

**Fig. 8:**
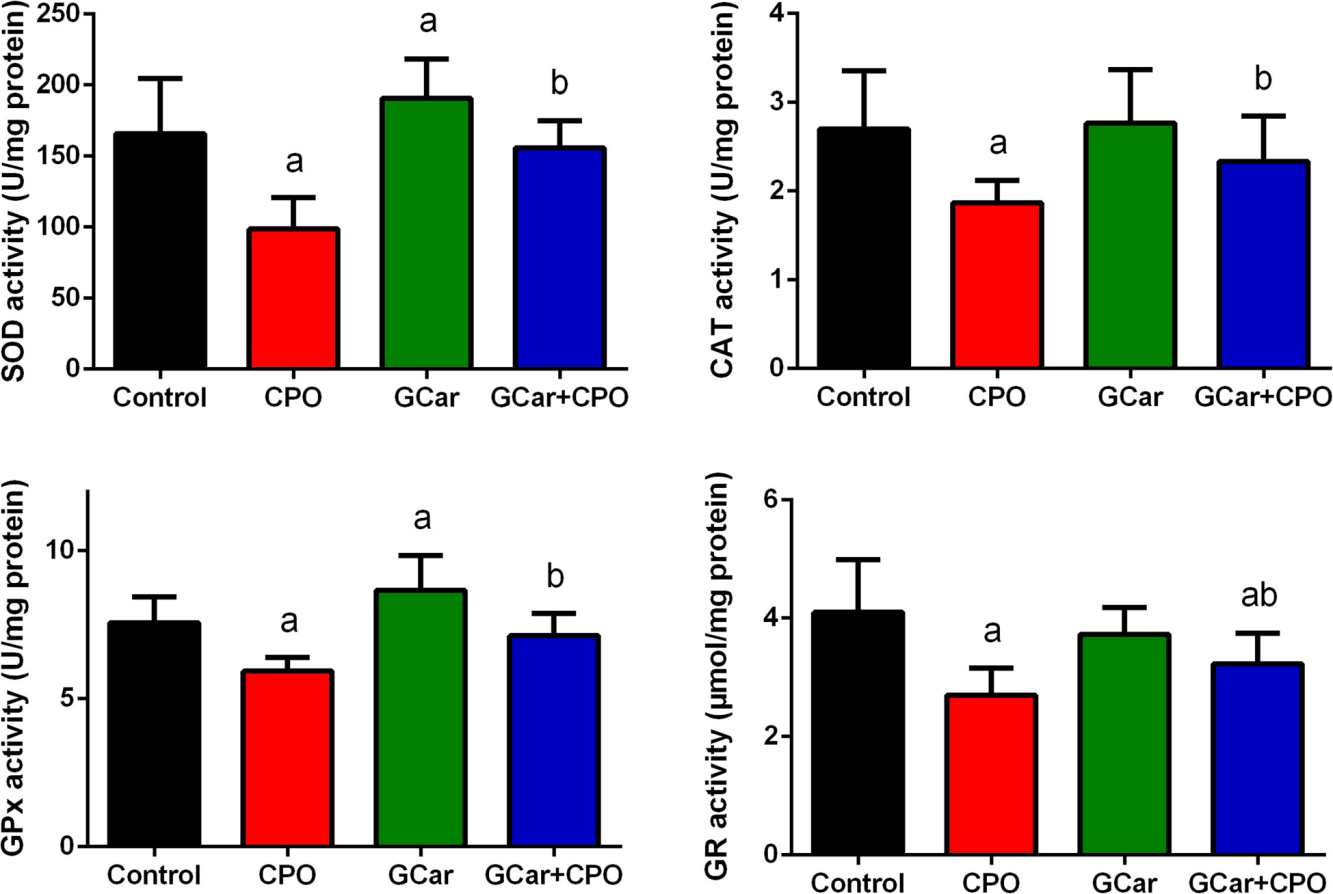
Effects of CPO, GCar and GCar-CPO on the SOD, CAT, GPx and GR activities in the tumor tissue. Values are expressed as means ± SD. a and b: Significant against control and CPO groups at *p* < 0.05, respectively

A significant rise in SOD and GPx activities in EST-treated groups with GCar treatment alone was recorded in comparison to its level in the EST control group. On contrary, there was a marked reduction in antioxidant enzyme activities in the EST–CPO-treated mice in comparison to its levels in the EST-control mice (Fig. 8). Additionally, treatment of EST bearing mice with GCar and GCar-CPO exhibited a marked increase in antioxidant enzyme activities compared to that of CPO treatment alone.

### Liver and kidney functions results

**Fig. 9:**
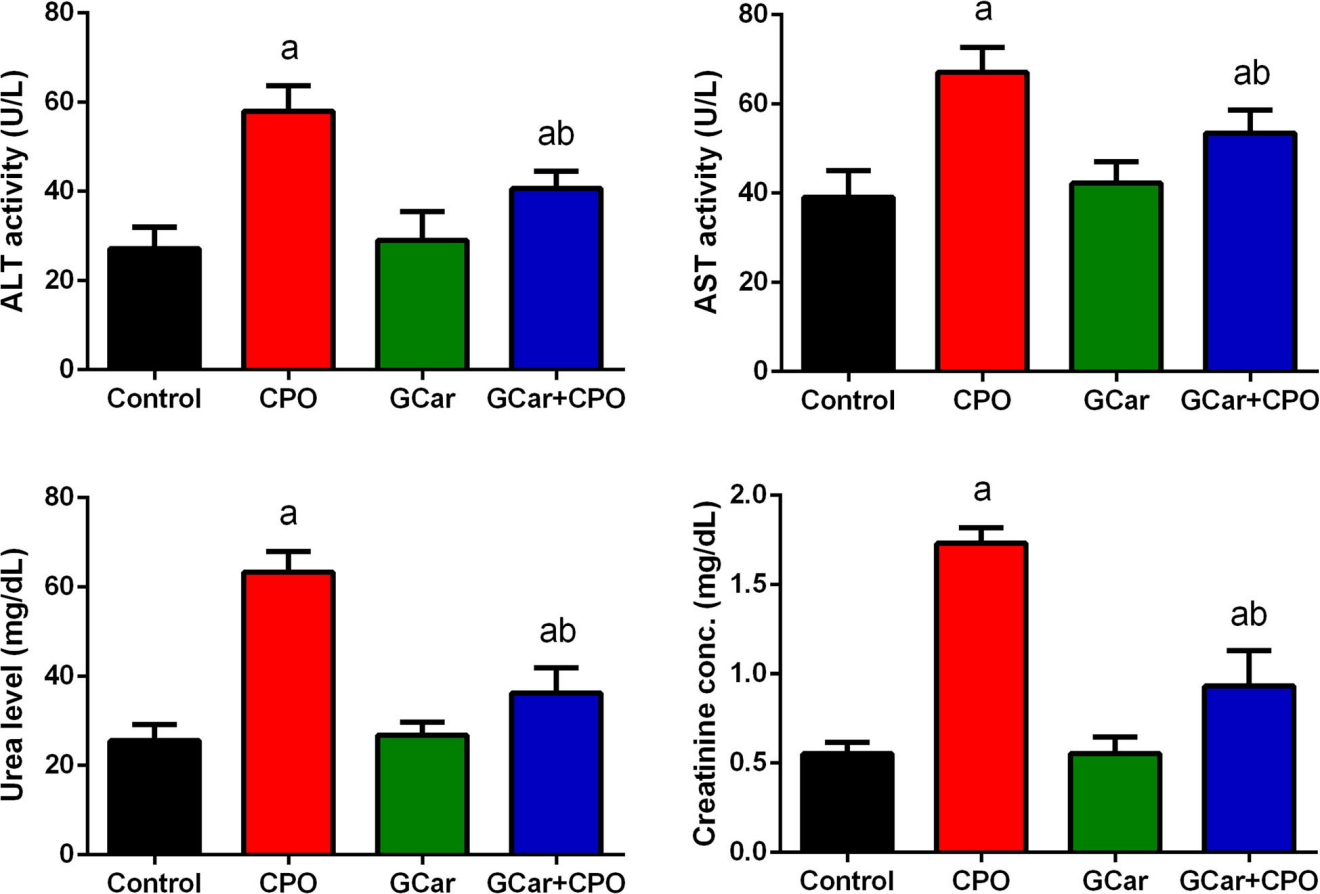
Effects of CPO, GCar and GCar-CPO on the ALT and AST activities and urea and creatinine levels in the serum. Values are expressed as means ± SD. a and b: Significant against control and CPO groups at *p* < 0.05, respectively

A non-significant change in liver and kidney function biomarkers were recorded in EST-treated groups with GCar treatment alone. However, there was a marked raise in those biomarkers observed in the CPO-treated mice in comparison to levels in the EST-control mice. On contrary, treatment of EST-bearing mice with GCar combined with CPO exhibited a marked decline in these biomarkers compared to those of the CPO treatment group alone (Fig. 9) suggesting the protective effect of cardamom against CPO.

## Discussion

The findings of the current investigation showed the importance of GCar as an antitumor agent obtained from natural sources whether used alone or combined with an anticancer drug. The anticancer agent suppressed the propagation of tumor cells by enhancing apoptosis and may represent a beneficial mechanistic strategy to both cancer chemoprevention and chemotherapy [28]. The results revealed that GCar displayed antitumor effect against EST tumors in mice, exhibited by the reduction in tumor weight and volume. The decline in body weight gain and diminish in tumor volume are the criteria for judging the efficacy of any anti-tumor drugs [5].

Growing body of evidences indicate that medicinal plants can modulate various molecular pathways involved in tumor initiation and growth. It is expected that researches with natural products will discover a diversity of molecular mechanisms and targets for tumor growth inhibition, apoptosis and especially angiogenesis prevention. To date, clinical trials in chemoprevention using medicinal products conducted for cancer are very limited. Extensive clinical study is warranted to assess further safety and chemo-preventive efficiency of natural products either alone or in combination with chemotherapeutic drugs against tumor [5].

Therapeutic molecules that targeted the cell death signaling, including pro-apoptotic proteins stimulators or suppressors of anti-apoptotic protein, could conquer the dilemma of resistance through directing the cancerous cells to self-destruct [29]. In this context, the examination of novel targeted apoptotic enhancers for cancerous cells could present a new approach in tumor treatment [5]. Accordingly, the goal of the present investigation was to study the apoptotic stimulating effect of GCar on cancer cells relative to their effects on the expression of pro-apoptotic and anti-apoptotic genes, as well as to assess the anticancer potential of GCar against a selected reference anticancer drug, CPO, in an in vivo EST model.

DNA is the principal target in terms of the teratogenic, mutagenic and antineoplastic effects of CPO. Effects of CPO on DNA have been extensively reported in mammalian cells, both of somatic and germ cell origins [30]. CPO is supposed to bring its cytotoxicity via the cross-linking of somatic DNA and researchers have found that following CPO treatment there is an occurrence of inter-strand and DNA-protein cross-links, but no single strand breaks [31]. CPO suppresses embryonic DNA biosynthesis and does so prior to its effect on RNA or protein construction [32]. Although CPO is known to cause DNA cross-links, other DNA damage is produced as well. CPO mediates G0/G1 and S phase arrest [33], accumulation of cells in the G0/G1 phase in comparison to that of the control, whereas higher amounts of CPO causes dose-dependent G0/G1, S and G2/M phase inhibition [34].

The cytotoxic action of CPO is induced mostly because of PM, which causes DNA cross-linking [35]. Alkylation of DNA and proteins by CPO causes the creation of cross-links. Cross-links in transcriptional active regions of the DNA can cause a silence or arrest of the RNA polymerases, negatively affecting transcription elongation and genes expression [36]. PM is a bifunctional alkylating molecule that binds to the N-7 position of guanine [37], to the phosphate backbone of DNA [38]. Exposure of cells to PM results in the generation of a mixture of inter-strand, DNA-protein cross-links and enlargement of cells. PM devastates quickly dividing cells and stimulates the DNA double strand break marker, γH2AX and DNA repair in rat granulosa cells and neonatal ovaries [39]. Acrolein, on the contrary, is found to bind to proteins, form DNA adducts, generate basic sites and to induce DNA single-strand breaks (SSB) [40].

CPO causes overproduction of ROS, such as the superoxide anions and hydroxyl radicals, which depletes GSH content and suppresses the activities of antioxidant enzyme in renal tissue. ROS may lead to cellular damage and necrosis via several mechanisms, including peroxidation of cellular membrane phospholipids, proteins denaturation and DNA injury [41]. Studies show that CPO induces ROS generation in vivo, which is accountable for the severe unfavorable effects of CPO treatment, including liver and kidney toxicities, which in turn are restrained by supplementation with antioxidants [42].

In the current study, CPO increased levels of LPO and decreased the GSH content in Ehrlich solid tumor-bearing mice. The antitumor activity of CPO was accompanied with the increase of oxidative stress status in tumor tissue. In rats, CPO increased LPO and altered the -SH status of the tissues with simultaneous alterations in enzymatic antioxidant molecules in other studies [43]. GSH content and GR activity are significantly diminished after CPO therapy, whereas GPx and CAT showed a significant improvement in the current study.

CPO-induced production of ROS has also been implicated in its direct cellular toxicity [44]. Acrolein, a reactive aldehyde, is known to be the most toxic metabolite of CPO, through its ability to produce toxic ROS and consequently affect surrounding tissues. ROS have various effects, including suppression of a variety of enzymes, membrane and DNA damage and lipid peroxidation [45]. In this regard, CPO injury can be ameliorated by free radical scavengers [46]. Our study is in agreement with the findings of Kurauchi et al. who found that CPO-induced cardiac apoptosis involves several pathways, including oxidant damage, creation of intrinsic and extrinsic apoptotic cascades and endonucleases [47]. Interestingly, we demonstrated that the treatment with GCar showed a significant reduction in oxidative stress markers and activation of the endogenous antioxidant system that affected by EST, suggesting that counteracting oxidative stress is critical in avoiding the progress of EST. The obtained results are in conformity with previous studies on GCar that showed the antioxidant activity in diethylnitrosamine-induced hepatocellular carcinoma [48]. Also, the attenuation of oxidative stress by GCar was expressed in its ability to restrain lipid peroxidation due to the presence of polyphenols and flavonoids.

Placzek et al. [49] reported that Bcl-2 and Bcl-x_L_ anti-apoptotic proteins were minimally expressed following treatment with CPO. Furthermore, the caspase-3, − 8 and − 9 are over-expressed in the treated tumor cells and this agreed with the results of Schwartz and Waxman [50], suggesting that caspase-9 may mediate apoptosis induced by CPO and its induction could represent a novel approach to the effective treatment of malignant tumors. Moreover, the authors found that Bcl-2 over-expression blocked the cytotoxic actions of activated CPO but did not suppress the drug’s cytostatic actions. CPO caused S-phase cell cycle arrest was followed by alteration to an apoptotic pre-G1 state in wild-type 9 L cells. In contrast, Bcl-2-expressing 9 L cells concentrated in G2/M in response to CPO management [50]. In addition, this is in agreement with Yang et al. [51], who showed that CPO treatment induced the activation/cleavage of caspase-3 in A431 cells. As a result, CPO treatment can initiate both the extrinsic and intrinsic pathways of caspases activation. Likewise, CPO-induced caspase-3 in Leukemic T cells [52] and the authors reported that oxidative stress-induced nuclear translocation of apoptosis-inducing factor (AIF) and endonuclease G (EndoG) in 4-hydroperoxy-CPO (4-OOH-CY)-treated T cells might represent an alternative death pathway in the absence of caspase activity. Other antioxidants such as B12 and sitagliptin, have previously been shown to have antitumor activities [16].

CPO induced ROS overproduction in vivo, which is accountable to the severe unwanted side effects of CPO treatment, including liver and kidney toxicities, which in turn are depleted by antioxidants addition. However, understanding the expression of anti- and pro-apoptotic proteins and their relationship to the redox system in the cells, as well as the location of ROS production upon CPO therapy will offer precious insights into new approaches for minimizing CPO unwanted side effects without affecting its effectiveness [53].

GCar has a wide range of biological effects, including anti-diarrheal, anti-hypertensive, anti-arrhythmic, cholesterol lowering, anti-microbial and anti-inflammatory activities [17, 54–57]. In addition, GCar caused inhibition of tumor cell proliferation and the induction of apoptosis and the mechanism of antitumor action differs among cell lines [54–56]. The anticancer effect of GCar may be attributed to the presence many phenolic compounds such as gallic acid and caffeic acid. GCar possesses a strong antioxidant effect with a ability to restrain the formation of tumors in many cancer models. In this context, Elguindy et al. [48] found that GCar treatment markedly diminished the levels of α-feto protein, which revealed the antitumor effect of GCar against hepatocellular carcinoma. Furthermore, Majdalawieh and Carr [2] found that GCar markedly promotes the cytotoxic activity of natural killer cells, suggesting its potential anticancer effect and Shinjini Pal et al. [58] found that zinc oxide nanoparticles green synthesized by GCar induced apoptosis in HepG2 cells. Moreover, Bhattacharjee and Chatterjee [59] observed that eucalyptol, abioactive compound of GCar, bind to Cas-3 and stimulated its activity.

Several cancerous cell lines have been utilized to study the action of GCar and it has been shown to be efficient at relatively low doses on a number of cancers arising from leucocytes, liver, brain, skin, lung, GIT, bone, and breast [9, 54, 56]. GCar extracts significantly improve the cytotoxic effect of natural killer cells, representing its anticancer potential [2]. The mechanism of its antitumor effect is that GCar modulates a variety of transcription signaling, growth factors, protein kinases and inflammatory cytokines [60].

## Conclusion

In summary, the combined application of GCar with CPO resulted in a statistically significant diminish in cell survival in comparison with GCar or CPO treatment alone. Therefore, the cytotoxic potential of GCar can be amplified by combination with CPO. Furthermore, our finding indicated that GCar is able to initiate apoptosis in cancer cells and maintained the balance between oxidants and antioxidants molecules.

## Supplementary Information

**Additional file 1: Table S1.** Identification of phytochemical compounds by HPLC in green cardamom (*Elettaria cardamomum*) seeds.

## Data Availability

All relevant data are within the paper.

## Abbreviations

ALT: Alanine aminotransferase
AST: Aspartate aminotransferase
Bax: Bcl-2-like protein 4
Bcl-2: B-cell lymphoma 2
BW: Body weight
CAT: Catalase
CPO: Cyclophosphamide
EAC: Ehrlich Ascites carcinoma
EST: Ehrlich solid tumor
GCar: Green cardamom
GPx: Glutathione peroxidase
GR: Glutathione reductase
GSH: Glutathione
H&E: Hematoxylin and Eosin
i.m.: Intramuscular
ILS %: Increased in life span percent
LPO: Lipid peroxidation
MDA: Malonaldehyde
MST: Mean survival time
NO: Nitric oxide
PCR: Polymerase chain reaction
ROS: Reactive oxygen species
SOD: Superoxide dismutase
TNF: tumor necrosis factor
