# Supplementary material for: Effects of Green cardamom (Elettaria cardamomum Maton) and its combination with cyclophosphamide on Ehrlich solid tumors
Source: BMC Complement Med Ther. 2021 Apr 29;21:133. doi: 10.1186/s12906-021-03305-2 (PMC8086365; doi:10.1186/s12906-021-03305-2)
Supplement: Supplementary file 1 — Additional file 1: Table S1. Identification of phytochemical compounds by HPLC in green cardamom (Elettaria cardamomum) seeds. [file 12906_2021_3305_MOESM1_ESM.docx]

**Table S1:** Identification of phytochemical compounds by HPLC in green cardamom (*Elettaria cardamomum*) seeds.

|  | **Assignment** | **RT** | **Area under peak (%)** |
| --- | --- | --- | --- |
|  | Ferulic acid | 3.3 | 1.3 |
|  | Ferulic acid derivatives | 3.8 | 1.8 |
|  | Luteolin | 4.5 | 0.9 |
|  | Rutin | 13.9 | 13.2 |
|  | Catechin | 14.3 | 18.7 |
|  | Caffeic acid | 15.3 | 0.4 |
|  | Gallic acid | 26.3 | 0.1 |
|  | Gallic acid derivatives | 27.7 | 33.1 |
|  | Quercetin | 30.2 | 2.4 |
|  | Cinnamic acid | 31.4 | 0.2 |

RT: Retention time.
